# Onyx Cast Migration to the Right Ventricle Following Trans-arterial Embolization of a Cranial Dural Arteriovenous Fistula

**DOI:** 10.7759/cureus.50368

**Published:** 2023-12-12

**Authors:** Mohamed Habib, Geraint Morton

**Affiliations:** 1 Cardiology, Portsmouth Hospitals University NHS Trust, Portsmouth, GBR

**Keywords:** ct chest, onyx embolization, onyx copolymer, dural arteriovenous fistula (davf), right ventricular cardiac mass

## Abstract

We report a case of a middle-aged man who presented to the cardiology clinic with an incidental finding of a hyperdense lesion in the right ventricle (RV). He is an ex-smoker and had a low-dose CT chest as part of a screening program for early lung malignancy.

His medical history included a cerebellar hemorrhage in 2021 due to a ruptured dural arteriovenous fistula (dAVF). He was treated as an emergency with trans-arterial embolization using Onyx liquid embolic material (Medtronic, Fridley, MN).

The high-flow dAVF embolization was straightforward, with Onyx filling the arteriovenous (AV) shunt and draining the vein. The patient made a good recovery, and routine cerebral digital subtraction angiography (DSA) at three months confirmed the occlusion of the dAVF.

Cardiac migration of liquid embolic material used to treat AV shunts is uncommon and probably underreported as it can be asymptomatic, as in this case. Cardiac embolization should be suspected in patients with dense material in the RV and prior treatment with trans-arterial embolization.

## Introduction

Cardiac masses can be divided broadly into neoplastic (benign and malignant tumors) and nonneoplastic. Benign tumors include myxomas, lipomas, fibromas, and teratomas. Malignant tumors include metastases, angiosarcomas, fibrosarcomas, and rhabdomyosarcomas. Nonneoplastic masses include thrombi, valvular vegetations, crista terminalis, pericardial cysts, and devices used in cardiac interventions [1-3].

Dural arteriovenous fistulae (dAVF) are abnormal arteriovenous (AV) shunts lying in the intracranial or spinal dura without a nidus. Etiology may be idiopathic or related to previous venous thrombosis, neurosurgery, trauma, or local infection. Endovascular treatment options include trans-arterial and trans-venous embolization with coils and/or liquid embolic material. Some dAVFs require open surgical disconnection [4-6].

Onyx (Medtronic, Fridley, MN) is a liquid embolic material, an elastic polymer comprising ethylene-vinyl alcohol copolymer dissolved in dimethyl sulfoxide with micronized tantalum powder. Onyx polymerizes upon contact with blood. Onyx is classified as a liquid, nonadhesive, nonabsorbable, permanent embolic agent. The tantalum powder provides contrast for fluoroscopic visualization. The Food and Drug Administration (FDA) approved Onyx in 2005 for the treatment of AV malformations [7].

Migration of Onyx casts is uncommon or an underreported occurrence [8].

## Case presentation

This case relates to a gentleman in his late sixties with a history of cerebellar hemorrhage and trans-arterial embolization of a dAVF in 2021. He is an ex-smoker and had a low-dose CT chest as part of a screening program to assess for early malignancy.

He was referred to the cardiology clinic after the low-dose CT chest identified an incidental finding of a hyperdense lesion in the right ventricle (RV) attached to the interventricular septum (IVS).

The patient was fit, active, and asymptomatic. He did not have chest pain, shortness of breath, syncope, or presyncope. He had no recurrence of headaches following embolization. He had a history of diabetes but no prior cardiac medical procedures. His clinical examination was entirely normal.

Investigations

The electrocardiogram (ECG) showed normal sinus rhythm at 67 beats per minute. His echocardiogram study had a poor image quality due to body habitus but no abnormalities were detected. Specifically, the RV was normal in size and function, and no mass was visible. There was no interventricular shunt and no evidence of pulmonary hypertension.

**Figure 1:**
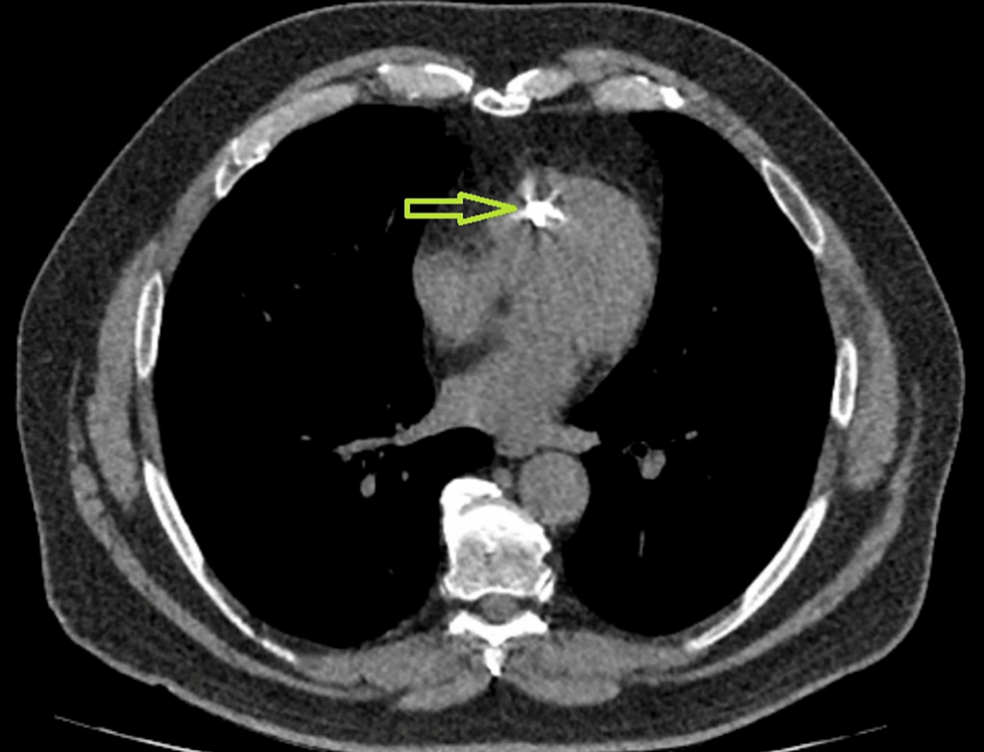
An axial section of the non-contrast CT chest, with an arrow pointing to the dense mass attached to the interventricular septum. CT, computed tomography

**Figure 2:**
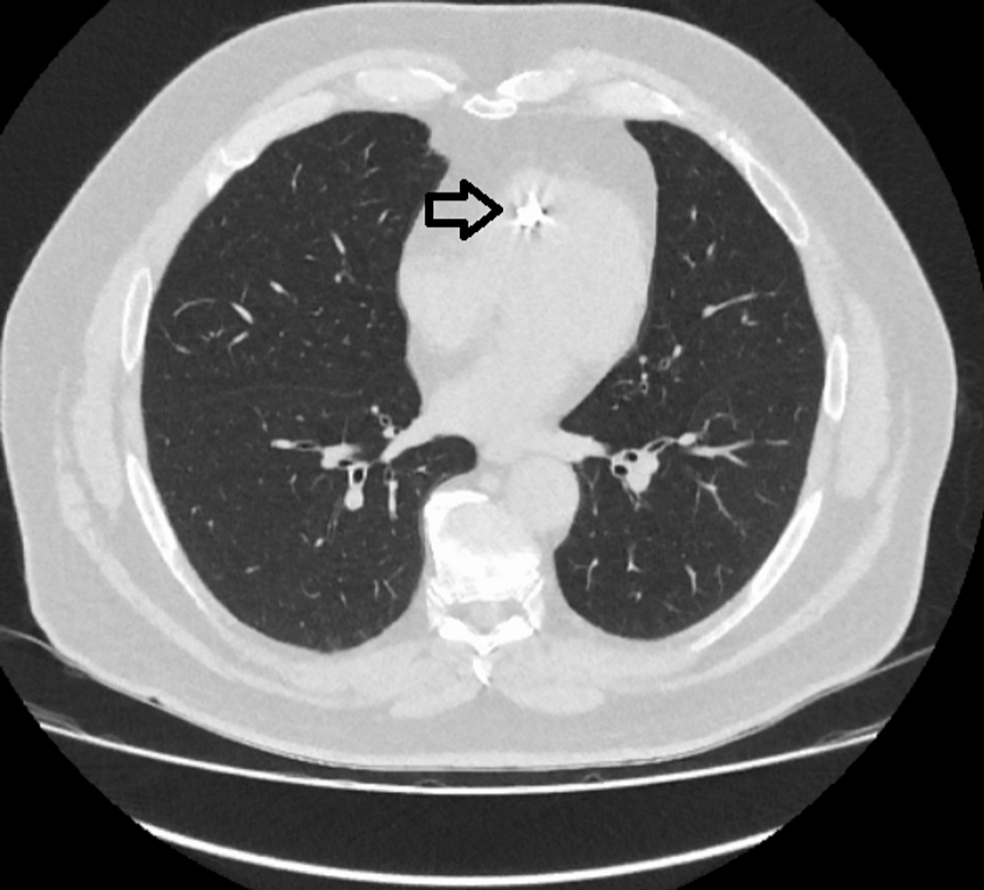
The lung window of the CT chest, with an arrow pointing to the bright dense mass. CT, computed tomography

**Figure 3:**
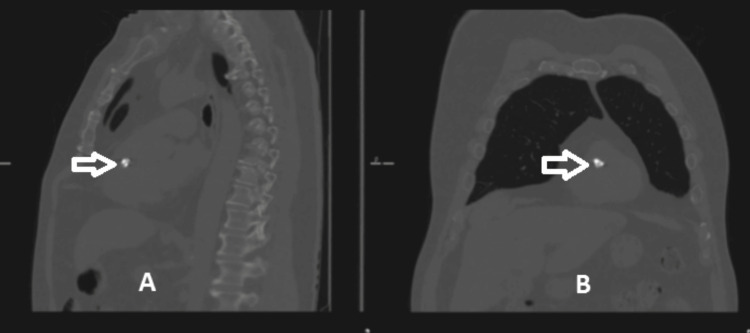
(A) A sagittal cut of the CT chest, with an arrow pointing to the dense mass; (B) a coronal cut of the CT chest, with an arrow pointing to the dense mass. CT, computed tomography

**Figure 4:**
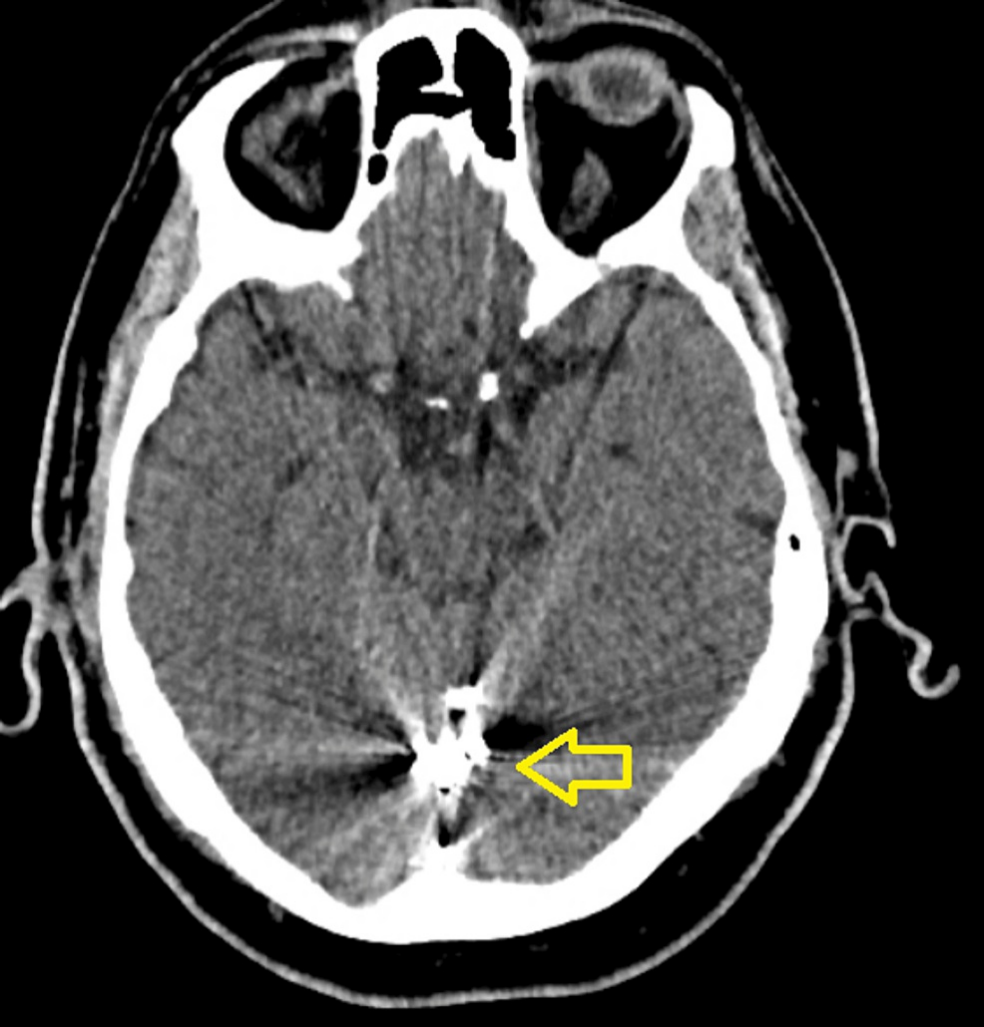
An axial cut of the non-contrast CT head, with an arrow pointing to the dense Onyx cast on the posterior cranial fossa. CT, computed tomography

**Figure 5:**
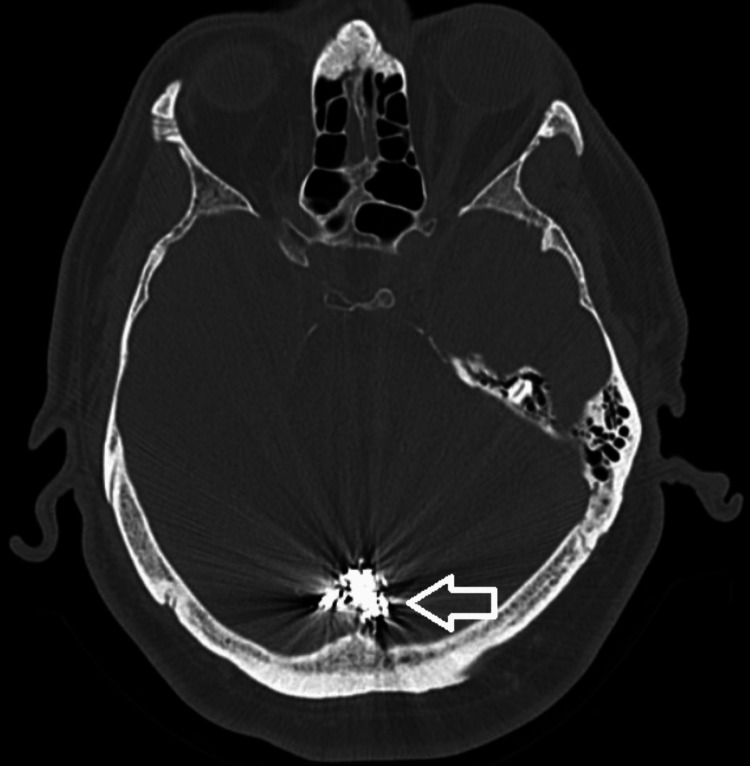
An axial cut of the CT head (bone window), with an arrow pointing to the dense Onyx cast in the posterior fossa. CT, computed tomography

The CT chest did not show lung nodules. However, there was an incidental finding of dense material with associated beam hardening in the RV adjacent to the IVS (Figures 1-3), and no dense material was visible in the pulmonary arteries. We arranged for a CT head, which showed a single dense Onyx cast in the posterior cranial fossa, similar in density to the material observed in the RV on the CT chest (Figures 4-5).

## Discussion

Migration of Onyx casts is an uncommon or underreported occurrence. In a case series by Wang et al. in Beijing-Titanium Hospital, it was reported that two patients with dAVF involving the transverse-sigmoid sinus were treated by trans-arterial embolization via the occipital artery and the posterior branch of the middle meningeal artery, respectively. A piece of Onyx was found in the RV on post-embolization chest X-ray film in both patients [8].

Our patient presented with a thunderclap headache in June 2021. He underwent a CT head, which revealed a cerebellar hemorrhage. Subsequent CT angiography and DSA confirmed a high-flow posterior fossa dAVF and demonstrated arterial supply and venous drainage.

There was no embolization of Onyx visible during procedural fluoroscopy screening. A retrospective review of DSA acquisitions did not demonstrate any change of the onyx cast to suggest embolization from the main cast. Presumably, there was early embolization of some Onyx cast from the dAVF draining vein, passing to the right ventricle where the cast became adherent to the wall.

Since Onyx casts are polymerized after contact with blood, it is likely to be attached to the interventricular septum and will not cause further embolization. Given that the procedure was done more than 24 months before the CT chest, the patient was asymptomatic, and based on our knowledge of right-sided cardiac devices, we opted not to start anticoagulation or antiplatelets. 

Whether Onyx could induce thrombosis and pulmonary embolization is unclear and requires further research. However, typically this does not occur with cardiac devices deployed in the RV, such as pacemakers.

## Conclusions

In patients with a history of embolization of AV fistulae or AV malformations, the potential migration of liquid embolic casts should be kept in mind. Liquid embolic casts, in the case of Onyx, appear hyperdense on CT scans because of the inclusion of tantalum powder.

Clarification is needed on whether migrated liquid embolic casts are at risk of further embolization, thus requiring treatment.
